# Envenimation scorpionique compliqué d'un accident vasculaire cérébral ischemique

**DOI:** 10.11604/pamj.2015.20.315.5604

**Published:** 2015-03-31

**Authors:** Youssef Bibiche, Adnan Berdai, Smael Labib, Mustapha Harandou

**Affiliations:** 1 Service de Réanimation Mère-enfant, Centre Hospitalier Universitaire Hassan II, Fès, Maroc

**Keywords:** Envenimation scorpionique, enfant, accident vasculaire cérébral ischémique, tomodensitométrique cérébrale, scorpion envenomation, child, ischemic stroke, brain computed tomography

## Abstract

Les piqûres de scorpion sont dans un certain nombre de pays, un accident fréquemment rencontré, et un risque grave. Au Maroc, les données épidémiologiques établies par le centre anti-poison (CAPM) montrent que les piqûres scorpioniques se placent en tête de toutes les intoxications relevées par le centre (60%). Les envenimations scorpioniques posent un grand problème de santé publique. L'apparition de signes neurologiques lors d'une envenimation scorpionique témoigne d'une envenimation grave et correspond au stade III de l’échelle de sévérité. Nous rapportons le cas d'une envenimation scorpionique compliqué d'un Accident vasculaire cérébral ischémique (AVCI) chez un enfant.

## Introduction

L'envenimation scorpionique est un véritable problème de santé publique en Afrique du Nord. Au Maroc, les piqûres scorpioniques se placent en tête de toutes les intoxications (60%), avec un taux de létalité globale de 0,82%, pouvant atteindre 5,3% dans certaines régions [1]. La survenue d'une envenimation chez l'enfant constitue un facteur de mauvais pronostic. Ainsi, selon le centre anti-poison du Maroc, le nombre de décès secondaire à une envenimation scorpionique chez l'enfant d'un âge inférieur à 15 ans est de 47 décès/an, par rapport à un taux de décès global à 55 décès/an [2]. L'accident vasculaire cérébral ischémique (AVCI) est une complication rare, notamment chez l'enfant, et témoigne d'une envenimation grave. Nous rapportons le cas d'une envenimation scorpionique compliqué d'un AVCI chez un enfant hospitalisé dans notre structure.

## Patient et observation

**Figure 1:**
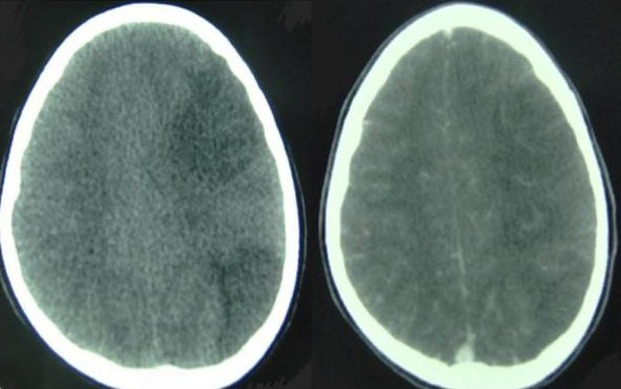
Présence d'une lésion hypodense du territoire de l'artère sylvienne superficielle gauche

Un enfant âgé de 12 ans, sans antécédent particulier, s'est dit victime d'une piqûre de scorpion noire au niveau de l'indexe de la main droite. 4 heures après, l'enfant a eu des sueurs, des douleurs abdominales, des vomissements et un priapisme. L'examen clinique à son admission révélait un enfant conscient, agité, eupnéique, apyrétique, stable sur le plan hémodynamique, sa pression artérielle est à 120 /70 mmHg et une frequence cardiaque a 110 b/min Après 12 heures d'hospitalisation, l'enfant a eu brutalement une hémiplégie droite avec participation faciale. L'examen neurologique a trouvé une hémiplégie droite, une paralysie faciale centrale, un signe de Babinski positif et des réflexes ostéotendineux abolis à droite. Image tomodensitométrique cérébrale objectivant un AVC ischémique du territoire de l'artère sylvienne superficielle gauche (Figure 1). L’électrocardiogramme (ECG) et L’échocardiographie sont anomalie. L’échographie des troncs supra-aortiques était normale et le dosage de la troponine était positif à 0,3. Le reste du bilan biologique était normal, avec absence d'anomalie de l'hémostase. Après une prise en charge symptomatique avec un remplissage vasculaire par du sérum salé 0,9% et l'administration d'antiémétiques et d'antalgiques, l’évolution de notre patient a été marquée par la récupération du déficit moteur au niveau du membre inférieur puis le membre supérieur jusqu’à la disparition totale, la persistance d'une légère paralysie faciale droite, et la négativation de la troponine Ic vers le 12^e^jour.

## Discussion

Les piqûres de scorpion sont dans un certain nombre de pays, un accident fréquemment rencontré, et un risque grave [1]. Les envenimations scorpioniques posent un grand problème de santé publique [3]. Au Maroc, les données épidémiologiques établies par le centre anti-poison (CAPM) montrent que les piqûres scorpioniques se placent en tête de toutes les intoxications relevées par le centre (60%) avec un taux d'incidence allant de 0 à 2,4% selon les régions et un taux de létalité globale de 0,82%, pouvant atteindre 5,3% dans les régions du Sud [4, 5]. Le taux de mortalité infantile entre l'année 1999 et 2008 était à 100 décès en moyenne [2].

Les toxines du venin de ces scorpions sont essentiellement neurotoxiques, elles agissent sur les canaux sodiques des cellules excitables, prolongent l'ouverture du canal sodique et tendent à entraîner une dépolarisation durable. C'est une stimulation neuronale de type présynaptique qui entraîne une libération massive de neuromédiateurs suivie d'un blocage de la transmission [4]. L'apparition de signes neurologiques lors d'une envenimation scorpionique témoigne d'une envenimation grave et correspond au stade III de l’échelle de sévérité [6, 7].

Les accidents vasculaires cérébraux sont parmi ces complications rares et pourraient être expliqués par la perturbation de la coagulation, le troubles du rythme et l'embolie systémique, l'hypotension, la dépression myocardique, l’état de choc [6, 8–10]. Chez notre patient, la symptomatologie était initialement des sueurs profuses, un priapisme et des douleurs abdominales. Par la suite il a eu brutalement une hémiplégie. Ce qui permet de classer son envenimation en classe III de sévérité [6]. L'AVCI est une complication grave pouvant mettre en jeu le pronostic vital et fonctionnel chez l'enfant [6].

## Conclusion

La prise en charge de l'envenimation scorpionique au Maroc est essentiellement symptomatique et comporte, l'administration des drogues vasoactives (dobutamine) en cas d’état de choc, l'oxygénothérapie et la ventilation artificielle en cas de détresse respiratoire et l'administration d'anticonvulsivant en cas de crises convulsives. Le traitement spécifique par l'immunothérapie antiscorpionique est très controversé n'est pas recommandée au Maroc. Une prise en charge symptomatique adéquate et une surveillance en milieu de soins intensifs ont permis une évolution très favorable de notre patient.
